# The effects of nocturnal hemodialysis compared to conventional hemodialysis on change in left ventricular mass: Rationale and study design of a randomized controlled pilot study

**DOI:** 10.1186/1471-2369-7-2

**Published:** 2006-02-22

**Authors:** Michael Walsh, Braden J Manns, Scott Klarenbach, Robert Quinn, Marcello Tonelli, Bruce F Culleton

**Affiliations:** 1 Department of Medicine, University of Calgary, Calgary, Canada; 2 Institute of Health Economics, Edmonton, Canada; 3 Department of Community Health Sciences, University of Calgary, Calgary, Canada; 4 Department of Medicine, University of Alberta, Edmonton, Canada; 5 Department of Medicine, Sunnybrook and Women's Health Sciences Centre, Toronto, Canada; 6 Department of Critical Care, University of Alberta, Edmonton, Canada

## Abstract

**Background:**

Nocturnal hemodialysis (NHD) is an alternative to conventional three times per week hemodialysis (CvHD) and has been reported to improve several health outcomes. To date, no randomized controlled trial (RCT) has compared NHD and CvHD. We have undertaken a multi-center RCT in hemodialysis patients comparing the effect of NHD to CvHD on left ventricular (LV) mass, as measured by cardiac magnetic resonance imaging (cMR).

**Methodology/design:**

All patients in Alberta, Canada, expressing an interest in performing NHD are eligible for the study. Patients enrolled in the study will be randomized to either NHD or CvHD for a six month period. All patients will have a full clinical assessment, including collection of biochemical and cMR data at baseline and at 6 months. Both groups of patients will be monitored biweekly to optimize blood pressure (BP) to a goal of <130/80 mmHg post-dialysis using a predefined BP management protocol. The primary outcome is change in LV mass, a surrogate marker for cardiac mortality, measured at baseline and 6 months. The high sensitivity and reproducibility of cMR facilitates reduction of the required sample size and the time needed between measures compared with echocardiography. Secondary outcomes include BP control, anemia, mineral metabolism, health-related quality of life, and costs.

**Discussion:**

To our knowledge, this study will be the first RCT evaluating health outcomes in NHD. The impact of NHD on LV mass represents a clinically important outcome which will further elucidate the potential benefits of NHD and guide future clinical endpoint studies.

## Background

Despite advances in dialysis therapy, mortality and morbidity remain high for patients with end-stage renal disease (ESRD). For instance, the mortality of this very high-risk population remains at nearly 20% per year with death rates from cardiac disease that are 10–20 fold higher in dialysis patients than in the general population [1]. Only 16% of incident dialysis patients have normal hearts by echocardiography, with concentric left ventricular hypertrophy (LVH) present in 41% and systolic failure in 16% [2]. This is important since the presence of LVH is associated with a several fold increase in risk for both heart failure and mortality [2,3].

Randomized trials that have attempted to demonstrate reduction in mortality among dialysis patients have generally been unsuccessful [4-8]. Even trials designed to impact surrogate endpoints, such as regression of LVH or LV dilatation, have been disappointing. Furthermore, there are very few interventions that have been shown to improve the poor health-related quality of life (HRQOL) observed in this population.

This difficulty of improving health outcomes may be due to the fact that ESRD is characterized by numerous complex metabolic and physiological abnormalities. Conventional hemodialysis (CvHD) does not normalize the majority of these abnormalities, and provides only a small fraction small molecular weight solute clearance compared with native kidney function[9]. In an attempt to improve urea clearance and clinical outcomes, nocturnal hemodialysis (NHD), a technique first developed in the 1970s, has received renewed attention. In NHD, patients perform dialysis at home; this is done five to six nights per week while they sleep.

Case control and cohort studies suggest that nocturnal hemodialysis may induce regression of LVH [10-12], improve HRQOL [13-15], and improve blood pressure control [16-18], among other health benefits. However, observational data may lead to conclusions ultimately refuted by randomized clinical trials (RCT). Given the uncertainty associated with the observed outcomes and costs derived from non-randomized studies examining NHD [19,20], it is important to have high quality data on NHD before widespread uptake of this new therapy occurs. We describe a randomized pilot trial comparing NHD (5 or 6 nights per week, 8 hours per night) and conventional hemodialysis (three times per week, four hours per session) with respect to their effect on progression of left ventricular mass measured by cardiac magnetic resonance imaging (cMR). Data on health-related quality of life, and measures of physiologic control, including blood pressure, anemia, calcium and phosphate metabolism and costs will be assessed as secondary outcomes.

## Methods/design

This study will be a prospective, two-center, parallel-group randomized, controlled study, with blinding of outcome assessors and data analysts. Approval has been granted by the local bioethics committees at the University of Calgary and the University of Alberta.

Eligible patients are men or women greater than 18 years of age who are currently receiving any hemodialysis modality (in-center, self-care or home) excluding current NHD. To be enrolled in this study, patients must be interested in and willing to train for NHD. Exclusion criteria include the inability to perform NHD due to physical or mental incapacity or the inability to provide informed consent.

Those patients meeting the inclusion and exclusion criteria will be randomized to either the NHD or control group. Randomization will be performed using a computer generated sequence in blocks of four patients. Randomization will be stratified by center and by baseline dialytic modality (self-care/home hemodialysis vs. in-center hemodialysis). Concealment will be assured through the use of sealed, opaque envelopes. Given the logistics of training patients within a nocturnal dialysis program, randomization will occur at one time point for patients willing to enter the study; however, patient entry into the study (i.e. the baseline study visit) will be staggered over time to facilitate NHD training in an orderly fashion. This will create a similar number of intervention and control patients at any given time point. This approach will be used to avoid an imbalance in drop-out rates between the two groups. Subjects randomized to CvHD will be eligible to commence NHD after study exit (six months).

### Objectives

The primary objective of this study is to evaluate the impact of NHD on the change in LV mass compared to CvHD in ESRD patients. LV mass will be measured by cMR at baseline and after six months of study and the difference will be compared between groups.

Secondary objectives of this study include the evaluation of NHD, as compared to CvHD, on (1) blood pressure, (2) HRQOL, (3) anemia, (4) mineral metabolism, and (5) health care and non-health care-related costs.

### Procedures – General considerations

Two centers in Alberta, Canada, the University of Calgary and the University of Alberta, will actively participate in the study. The first patient was enrolled in August 2004 and by January 2006 enrollment is expected to be complete. All patients will be followed for a period of six months for the primary and secondary objectives. Given the nature of the intervention, it is not possible to blind patients and investigators to the study intervention; however, the radiologists assessing the cMR studies (i.e. outcome assessors) and data analysts will be blinded to patient allocation.

Formal study visits will occur at study entry and at six months (study exit). These will be performed by physicians involved in the study and will be comprised of questionnaires, standardized blood pressure measurements, and reviews of recent dialysis sessions and laboratory work (see Table 1). Entry and exit visits will be performed within one week of entry and exit cMR tests. The baseline study visit will occur immediately pre-dialysis on a mid-week dialysis day for both groups. Exit study visits will occur immediately pre-dialysis on a mid-week dialysis day for the CvHD patients and ~9 hours post-dialysis (~5 hours predialysis) for NHD patients.

**Table 1:** Data collection time points.

Data Collected	Month of Study							
	Randomization	Baseline	1	2	3	4	5	6

Baseline Characteristics		**X**						**X**
Comorbidity Data		**X**						
cMR		**X**						**X**
Blood Pressure		**X**						**X**
HRQOL	**X**	**X**						**X**
Hbg/Hct		**X**	**X**	**X**	**X**	**X**	**X**	**X**
Erythropoeitin		**X**	**X**	**X**	**X**	**X**	**X**	**X**
Calcium/Phosphate		**X**	**X**	**X**	**X**	**X**	**X**	**X**
PTH		**X**						**X**
Medications		**X**	**X**	**X**	**X**	**X**	**X**	**X**
Kt/V		**X**						**X**
Economic Data		**X**						**X**

### Procedures – Nocturnal hemodialysis

**Figure 1:**
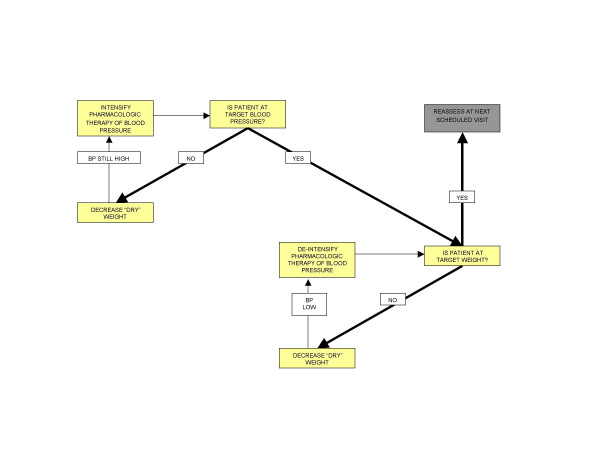
Blood pressure management algorithm.

Patients will initially be trained in-center for four to five days per week with direct nursing supervision and monitoring of biochemical parameters. During this training period dry weight and medications will be adjusted as dictated by blood pressure and biochemical parameters. Target weight will be adjusted over the course of the first 4 weeks to achieve a goal postdialysis blood pressure of 130/80, with introduction and discontinuation of blood pressure medications according to the protocol outlined below and in Figure 1. Anemia management will be carried out according to a standardized nursing-led anemia protocol aiming for a goal hemoglobin of 110 – 120 g/L utilizing erythropoietic stimulating proteins and iron supplements as necessary [21]. Mineral metabolism management will be carried out by the patient's attending nephrologist according to local treatment goals (calcium < 2.55 mmol/L, phosphate <1.80 mmol/L and intact PTH 150 – 300 ng/L).

Upon completion of training, nocturnal hemodialysis will be performed by the patients, at home, without remote monitoring 5 – 6 nights per week for a minimum of 6 hours per night. Dialysis will be performed with blood flow rates of 250 ml/min in patients receiving dialysis by central venous catheter or 150–200 ml/min in patients dialyzing by a single needle, button-hole technique in a graft or fistula. Dialysate flow rates of 300 ml/min were used for all patients.

### Procedures – Conventional hemodialysis

Patients will continue their pre-randomization dialysis modality (in-center, self-care or home hemodialysis) and dialysis prescription. Blood pressure management will be standardized as outlined below and in Figure 1. Anemia and mineral metabolism management will be carried out as per the protocols noted above for the NHD patients.

### Procedures – Blood pressure treatment

Blood pressure and antihypertensive medication use are potentially important determinants of LVH. In an attempt to assess whether the hypothesized impact of NHD on LV mass is due to improved BP control or to a non-BP related effect, both arms of the study will be aggressively treated to a post-dialysis blood pressure goal of 130/80 mmHg. This goal will be achieved by implementation of a management algorithm (see Figure 1). Although there is controversy as to the goal BP in hemodialysis patients [22], and in addition, whether pre- or post- dialysis pressures correlate best with cardiovascular outcomes, for the purposes of this algorithm, the blood pressure of interest will be the recording obtained by the dialysis machine's automated blood pressure cuff immediately post-dialysis. Blood pressures will be reviewed and appropriate changes made as per the blood pressure algorithm every two weeks. Antihypertensive medications previously prescribed by the patients' nephrologist will be left in place at study entry. The protocol mandates stepwise reductions in dry-weight as tolerated by the patient before intensification of their pharmacologic routine. Pharmacologic intervention is preferentially accomplished by first increasing doses of already prescribed angiotensin-converting enzyme inhibitors or angiotensin receptor blockers followed by beta blockers or calcium channel blockers, and then other antihypertensives. The use of direct vasodilators will be avoided when possible. If these manipulations are not successful in achieving target blood pressure other agents may be added.

### Outcomes – Left ventricular mass

LV mass, in grams, will be measured by cMR at baseline and at month six for patients in both arms of the study. MR imaging will be performed in each patient using a 1.5-T system (Sonata Software versions Syngo 2002B and 2004A; Siemens Medical Solutions, Erlangen, Germany). ECG gating will be used for all acquisitions. Briefly, routine localizers will be obtained and a four chamber localizer will be used. By using the end-diastolic cine frame of this four-chamber view, a series of parallel short-axis image planes can be defined, starting at the base of the LV and right ventricle (RV) and encompassing the entire LV and RV from base to apex. The most basal image plane will be positioned well into the atria. This will also ensure that the most basal part of the ventricles will be covered. The entire LV/RV will be covered with 10–12 slices (10 mm thick, gap 2 mm). At every short-axis plane, a breath-hold cine acquisition will be performed. For cine imaging, a steady-state free precession pulse sequence will be applied with 15 ky lines per heartbeat. Retrospective gating will be used with an acquisition window of 20% greater than the average RR interval and 20 phases will be calculated per RR interval. The field of view will be adapted to the individual patient to obtain optimal images. Data will be analyzed using vendor provided software (Argus, Siemens Medical Solutions, Erlangen, Germany). Endocardial, epicardial and papillary muscle outlines will be manually traced on end systolic and end diastolic frames. Ejection fraction, LV mass, and stroke volume will be calculated.

### Outcomes – Blood pressure

Assessment of the blood pressure outcomes will be performed at baseline and month six of the study by a study physician using a mercury sphygmomanometer following the CHEP protocol for blood pressure measurement [23]. A five-minute rest in a quiet, comfortable room will be used prior to measurement. Three blood pressure measurements will be taken five minutes apart with the average of the last two blood pressures used as the true blood pressure.

Blood pressure control will also be evaluated by the mean change in blood pressure parameters (systolic, diastolic, mean arterial, and pulse pressure). The use of antihypertensive medications will also be assessed at both the entrance and exit visit and the change in the mean number of antihypertensive medications will be compared between the NHD and CvHD group.

### Outcomes – Health related quality of life

Health-related quality of life questionnaires will be administered at the time of randomization (before patients are aware of their treatment allocation), at baseline (i.e. month 0), and at three and six months of the study. Consistent with published guidelines [24], HRQOL will be assessed using a spectrum of HRQOL instruments including a disease specific questionnaire (Kidney Disease Quality of Life Short Form (KDQOL-SF), a generic questionnaire (Short-Form 36 (SF-36), and a preference-based questionnaire (Euroqol (EQ-5D)) [25,26].

The EQ-5D will be used in this study as a preference-based index measure to approximate utility scores. Questions in the EQ-5D index focus on five dimensions: mobility, self-care, usual activities, pain/discomfort and anxiety/depression. The EQ-5D Index, based on health state valuations elicited from the general public [27,28] is anchored at 0.0 (dead) and 1.0 (full health). A minimum increment of 0.03 in the EQ-5D index score can be considered clinically important as it corresponds to the smallest coefficient for a change from 'No problem' to 'Some problems' within a dimension of the EQ-5D (26). Given that the EQ-5D measures overall quality of life, the EQ-5D index score will be the primary measure used to determine whether NHD has an impact on HRQOL.

The KDQOL-SF includes questions targeted at particular health-related concerns for individuals on dialysis. Scores on each KDQOL-SF dimension range from 0 – 100, with higher scores reflecting better HRQOL. The generic HRQOL profile measure used in this study will be the SF-36, which has been used in ESRD [29] and includes eight multi-item scales describing general HRQOL issues. As with the KDQOL-SF, scores on each dimension can range from 0 – 100, with higher scores reflecting better HRQOL. These measures will predominantly be measured to determine which facets of HRQOL are most impacted by NHD.

### Outcomes – Anemia

Hemoglobin and hematocrit will be measured every month throughout the study. The dose of erythropoietic stimulating proteins and iron supplements will be recorded for two months prior to starting the study as well monthly for the duration of the study. The effect of the intervention on anemia management will be primarily assessed using the change in mean erythropoietin/hematocrit ratio. As well, since both groups may obtain similar hemoglobin levels by the use of erythropoietin and iron, the average doses of these medications for the two months before study entry and the last two months of the study will also be compared between groups.

### Outcomes – Mineral metabolism

Calcium, phosphate, and albumin levels will be measured monthly throughout the study. PTH levels will be drawn at baseline and study exit. Information on the dose and type of phosphate binder, and the dose, type and route of administration for vitamin D analogs will be recorded monthly throughout the study.

### Outcomes – Costs

To enable economic evaluation of NHD, use of health care resources and their associated costs will be collected prospectively alongside the clinical trial. Costs to be assessed will include the costs associated with NHD, including the cost of training patients, the cost of necessary renovations to a patient's home, the cost of the hemodialysis machine, and the ongoing cost of supplies and clinical support. The cost of providing dialysis for patients in the conventional hemodialysis group will be assessed as previously described [30]. In addition to collecting dialysis-specific costs, we will also collect information for all patients on associated health care costs including the cost of hospitalization, the cost of outpatient day surgery (including access surgery), the cost of diagnostic imaging (including that required to maintain the patient's vascular access), the cost of medications, and the cost of laboratory testing [30]. Lastly, patients will complete a questionnaire at study entry and exit to assess the non-health care related costs associated with performing nocturnal and conventional dialysis.

### Statistical considerations

This will be a superiority study undertaken to test the hypothesis that NHD leads to less LV mass progression compared to CvHD. Based upon observational data obtained by echocardiography (11), the baseline LV mass for both groups will be 147 +/- 8 g. To give the study the power (1-beta = 0.95) to detect a significant (alpha = 0.05, two-tailed test) 10 g difference in LV mass (a clinically important and statistically detectable difference given the use of CMR) between the 2 treatment groups, 32 patients are needed to complete the study. Assuming 20% dropout (due to transplantation or death over six months), 19 patients in each arm of the study will be enrolled.

All analyses will be undertaken by a data analyst blinded to the subject treatment assignment. Summary statistics will be used to describe the baseline characteristics of both groups. For the primary endpoint of change in LV mass, the intention-to-treat (ITT) approach will be used, including all enrolled patients who have at least one cMR measurement. The analysis of the primary end-point will be based on the difference between the groups in the change of LV mass over the study period. For patients who drop-out of the study (death or renal transplant) before the final cMR examination, we will assume no change in the baseline cMR parameters for the primary ITT analyses. This very conservative approach will likely bias the final results towards the null hypothesis. A similar approach will be used for the secondary endpoints (Table 2). Exploratory secondary analyses will be performed using an observed cases approach.

**Table 2:** Primary analyses for primary (LV mass) and secondary outcomes

Factor	First Measure	Final Measure	Primary Analysis*
LV mass	Baseline	6 month visit	Comparison of mean change in LV mass for NHD vs control subjects
Blood pressure	Baseline visit – average of 5 and 10 minute readings	6 month visit – average of 5 and 10 minute readings	Comparison of mean change in systolic BP for NHD vs control subjects
Quality of Life	Baseline	6 month visit	Difference in the 6 month and baseline EQ-5D index scores for NHD vs control group – intention to treat, last observation carried forward
Anemia	Mean Hct and Epo administered for two months preceding baseline visit	Mean Hct and Epo administered for the final two months of the study	Mean change in hct/epo ratio for NHD vs control subjects
Mineral Metabolism	Mean Ca*PO4 for two months preceding baseline visit	Mean Ca*PO4 for the final two months of the study	Mean change in Ca*PO4 for NHD vs control subjects

*All randomized subjects with at least one measurement are included.

## Discussion

Previous observational studies have reported regression of LV mass with the use of NHD. Given that LV mass is a surrogate measure of cardiovascular disease and is an independent predictor of cardiac mortality [2,31], this is an important finding which has fostered enthusiasm for NHD as a standard offering for dialysis patients. Observational studies have also reported other clinical benefits with NHD including improved blood pressure control and serum phosphate. Despite these notable advantages, NHD has not yet been compared to CvHD in a randomized controlled trial. This is of paramount importance as the effort and cost required to implement NHD as a standard dialytic therapy may be imposing. In addition, several interventions leading to improved health outcomes in observational studies of dialysis patients, have failed the "gold standard" of showing the same improvements in randomized controlled trials [4,5,7].

Although this study will not be powered to detect a difference in mortality, the study design will permit conclusions with respect to relevant outcomes, including LV mass progression, control of serum phosphate and anemia, and change in HRQOL. The study will also facilitate the design of a larger randomized trial which examines clinically relevant endpoints including mortality.

## Abbreviations

CHEP – Canadian Hypertension Education Program

cMR – cardiac magnetic resonance

CvHD – conventional hemodialysis

EQ-5D – Euroqol 5-D

ESRD – End-stage Renal Disease

HRQOL – health related quality of life

KDQOL-SF – Kidney disease quality of life short form

LV – left ventricle

LVH – left ventricular hypertrophy

NHD – nocturnal hemodialysis

RCT – randomized controlled trial

SF-36 – short form 36

## Competing interests

The author(s) declare that they have no competing interests.

## Authors' contributions

MW: participated in manuscript preparation, patient enrollment and study conduct and data collection.

BM: participated in manuscript preparation, acquisition of funding, study design, patient enrollment and study conduct and data collection.

SK: participated in manuscript preparation, patient enrollment and study conduct and data collection.

RQ: participated in manuscript preparation and study design.

MT: participated in manuscript preparation

BC: participated in manuscript preparation, acquisition of funding, study design, patient enrollment and study conduct and data collection.

All authors read and approved of the final manuscript.

## Pre-publication history

The pre-publication history for this paper can be accessed here:


